# Risk profiling of soil-transmitted helminth infection and estimated number of infected people in South Asia: A systematic review and Bayesian geostatistical Analysis

**DOI:** 10.1371/journal.pntd.0007580

**Published:** 2019-08-09

**Authors:** Ying-Si Lai, Patricia Biedermann, Akina Shrestha, Frédérique Chammartin, Natacha à Porta, Antonio Montresor, Nerges F. Mistry, Jürg Utzinger, Penelope Vounatsou

**Affiliations:** 1 Swiss Tropical and Public Health Institute, Basel, Switzerland; 2 University of Basel, Basel, Switzerland; 3 Department of Control of Neglected Tropical Diseases, World Health Organization, Geneva, Switzerland; 4 Foundation for Medical Research, Mumbai, Maharashtra, India; Universidade Federal de Minas Gerais, BRAZIL

## Abstract

**Background:**

In South Asia, hundreds of millions of people are infected with soil-transmitted helminths (*Ascaris lumbricoides*, hookworm, and *Trichuris trichiura*). However, high-resolution risk profiles and the estimated number of people infected have yet to be determined. In turn, such information will assist control programs to identify priority areas for allocation of scarce resource for the control of soil-transmitted helminth infection.

**Methodology:**

We pursued a systematic review to identify prevalence surveys pertaining to soil-transmitted helminth infections in four mainland countries (i.e., Bangladesh, India, Nepal, and Pakistan) of South Asia. PubMed and ISI Web of Science were searched from inception to April 25, 2019, without restriction of language, study design, and survey date. We utilized Bayesian geostatistical models to identify environmental and socioeconomic predictors, and to estimate infection risk at high spatial resolution across the study region.

**Principal findings:**

A total of 536, 490, and 410 georeferenced surveys were identified for *A*. *lumbricoides*, hookworm, and *T*. *trichiura*, respectively. We estimate that 361 million people (95% Bayesian credible interval (BCI) 331–395 million), approximately one-quarter of the South Asia population, was infected with at least one soil-transmitted helminth species in 2015. *A*. *lumbricoides* was the predominant species. Moderate to high prevalence (>20%) of any soil-transmitted helminth infection was predicted in the northeastern part and some northern areas of the study region, as well as the southern coastal areas of India. The annual treatment needs for the school-age population requiring preventive chemotherapy was estimated at 165 million doses (95% BCI: 146–185 million).

**Conclusions/significance:**

Our risk maps provide an overview of the geographic distribution of soil-transmitted helminth infection in four mainland countries of South Asia and highlight the need for up-to-date surveys to accurately evaluate the disease burden in the region.

## Author summary

Hundreds of millions of people in South Asia are infected with parasitic worms, such as hookworm, roundworm, and whipworm. However, precise information on where these infections occur and the exact number of people affected is not available. Such information though is important to aid control programs, so that interventions can be targeted to priority areas and limited financial and human resources allocated in a cost-effective manner. We did a systematic review of the literature to collect prevalence data on soil-transmitted helminth infections and used Bayesian geostatistical models to predict infection risk at high spatial resolution in four mainland countries (i.e., Bangladesh, India, Nepal, and Pakistan) of South Asia. These countries account for 97% of the population in the region. We estimate that more than 350 million people were infected with at least one species of parasitic worms in 2015. The risk maps provide an overview of the geographic distribution of parasitic worm infections in the study region. Our results highlight the need for up-to-date surveys to more accurately evaluate the disease burden in South Asia.

## Introduction

Soil-transmitted helminths (i.e., *Ascaris lumbricoides*, hookworm, and *Trichuris trichiura*) are widespread, particularly in resource-constrained settings and marginalized populations [1]. Indeed, soil-transmitted helminth infections are among the most prevalent of the neglected tropical diseases (NTDs), and they rank among the top three according to global prevalence and population at risk of all NTDs [2]. In 2010, it was estimated that 819 million people were infected with *A*. *lumbricoides*, 465 million with *T*. *trichiura*, and 439 million with hookworm [3], accounting for a global burden of 5.2 million disability-adjusted life years (DALYs) [4]. The regions with the highest prevalence of soil-transmitted helminth infection are East Asia, including the People’s Republic of China and the Pacific Islands, sub-Saharan Africa, South Asia, and Latin America and the Caribbean [1,5].

According to the World Bank, South Asia consists of six mainland countries; namely, Afghanistan, Bangladesh, Bhutan, India, Nepal, and Pakistan, and two island countries, the Maldives and Sri Lanka [6]. Four of these countries (i.e., Bangladesh, India, Nepal, and Pakistan) account for 97% of the population in South Asia. Even though regional economic growth in South Asia was projected to increase according to a World Bank report in 2019 [7], there is still a large number of people living in poverty. Indeed, in 2013, approximately 776 million people in Bangladesh, India, Nepal, and Pakistan lived on less than US$ 1.9 per day, which is considered the poverty line [8]. Moreover, South Asia still has the highest rates and largest numbers of malnourished children, which is improving only very slowly [9].

It was estimated that, in 2010, there were 298 million, 140 million, and 101 million individuals in South Asia infected with *A*. *lumbricoides*, hookworm, and *T*. *trichiura*, respectively, thus accounting for more than one-quarter of the world’s soil-transmitted helminth infections [3]. In 2001, the World Health Assembly (WHA) set the global target of regular deworming of at least 75% of school-age children at risk of soil-transmitted helminth infection by 2010 [10]. Periodic large-scale preventive chemotherapy is recommended by the World Health Organization (WHO) when prevalence in school-age children exceeds a pre-defined threshold [11]. Here, we consider that people living in communities where prevalence is above this threshold are those requiring preventive chemotherapy. Interestingly, a school-based national survey in Sri Lanka showed that the country had a prevalence of soil-transmitted helminth infections in 2003 below the WHO threshold warranting preventive chemotherapy [12]. Data from the WHO Preventive Chemotherapy and Transmission Control (PCT) databank showed that before 2010, only Bhutan achieved the target of preventive chemotherapy with coverage of at least 75% of school-age children at risk [13]. Bangladesh reached this target for the first time in 2012, Nepal in 2012/2013, India in 2015, and Afghanistan in 2016. For Pakistan and the Maldives, no data are currently available for drug coverage of school-age children from 2010 onwards. Information is lacking on infection risk of soil-transmitted helminths in the Maldives.

High-resolution, model-based risk maps depicting the geographic distribution of soil-transmitted helminth infection can assist disease control programs by helping governments and policy makers deliver and monitor preventive chemotherapy and other interventions. Large-scale risk estimates of soil-transmitted helminth infections have been generated for the People’s Republic of China, Latin America, and sub-Saharan Africa [14–16]. However, risk maps for soil-transmitted helminth infection are currently lacking for South Asia. Bayesian geostatistical modeling is a powerful approach to produce risk maps for NTDs, by relating disease survey data to potential risk factors, thus predicting infection risk in areas without observed data [17–19].

In this paper, we presented the first comprehensive risk estimates of soil-transmitted helminth infection in four countries of mainland South Asia; namely, Bangladesh, India, Nepal, and Pakistan. Despite considerable efforts, we only obtained little information on georeferenced soil-transmitted helminth infection survey data after 2000 in Afghanistan, Bhutan, the Maldives, and Sri Lanka, and hence, these countries were not included in our Bayesian geostatistical modeling [6,20].

## Methods

### Ethics statement

The work presented here was facilitated by prior surveys pertaining to soil-transmitted helminth infection, readily derived from the literature. All data in our study were aggregated at the unit of villages, towns, or districts, and did not contain information identifiable at individual or household level. Hence, there were no specific ethics issues that warranted special attention.

### Soil-transmitted helminth infection data

A systematic review was undertaken following the PRISMA guidelines [21]. We searched PubMed and ISI Web of Science from inception to April 25, 2019 for relevant publications that reported data of infection prevalence with any of the three common soil-transmitted helminth species in Bangladesh, India, Nepal, and Pakistan. The following search terms were utilized: helminth* (OR ascari*, OR trichur*, OR hookworm*, OR necator, OR ankylostom*, OR ancylostom*, OR geohelminth*, OR nematode*) AND South Asia (OR Bangladesh, OR India, OR Nepal, OR Pakistan). We also considered the grey literature (e.g., Ministry of Health reports or relevant documents from research groups, PhD theses, etc.). As we tried to identify all potentially relevant studies, we set no restriction for language of publication, date of survey, or study design in our search strategy. Further criteria were applied to exclude studies that were not fit for our analysis. A similar search strategy was also employed for Afghanistan, Bhutan, the Maldives, and Sri Lanka separately for each country.

With regard to inclusion, exclusion, and extraction of survey data, we followed the protocol put forth by Chammartin and colleagues [14]. In brief, we excluded case reports, *in vitro* investigations, non-human studies, and surveys that did not report soil-transmitted helminth infection prevalence data. We also excluded case-control studies, clinical trials, drug efficacy, or intervention studies (except for baseline data or control groups), or locations where preventive chemotherapy occurred within one year (if such information was mentioned in the corresponding literature), or studies done in specific groups that might not be representative (e.g., travelers, military personnel, expatriates, nomads, or displaced or migrating populations). As the current study systematically reviewed prevalence data mainly obtained from cross-sectional surveys rather than clinical trials, we did not consider publication bias or selective reporting bias. In our view, these sources are negligible because high or low prevalence estimates are less likely to influence the decision of researchers to publish or to select subsets of analyses to report.

Data were georeferenced and entered together with detailed survey information into the open-access Global Neglected Tropical Diseases (GNTD) database [22]. We adhered to our review protocol with clear inclusion, exclusion, and extraction criteria. Hence, the quality of our final included studies was high. We did not assess the quality of each individual study separately, as these studies were published in the peer-reviewed literature. As we did not assess interventions, we did not address item #20 in the PRISMA checklist. Our final analysis included data derived from surveys conducted from 1950 onwards, either school- or community-based, aggregated at village or town level, or on administrative divisions of level two or three (district level).

### Climatic, demographic, environmental, and socioeconomic data

Climatic, demographic, and environmental data were obtained from readily accessible data sources, as summarized in Table 1. Land surface temperature (LST) and normalized difference vegetation index (NDVI) were averaged over the period of 2000–2015, while land cover was summarized by the most frequent category over the period of 2001–2012. According to similar classes, land cover data were further re-grouped into seven categories; namely, (i) grasslands; (ii) forests; (iii) scrublands and savannas; (iv) croplands; (v) urban; (vi) wet areas (water bodies or permanent wetlands); and (vii) barren areas.

**Table 1:** Remote sensing data sources employed for the current systematic review pertaining to soil-transmitted helminth infection in South Asia^a^.

Source	Data type	Data period	Temporal resolution	Spatial resolution
MODIS/Terra^b^	LST^k^	2000–2015	8 days	1 km
MODIS/Terra^b^	NDVI^l^	2000–2015	16 days	1 km
MODIS/Terra^b^	Land cover	2001–2012	Yearly	500 m
WorldClim^c^	Elevation	2000	-	1 km
WorldClim^c^	Bioclimatic variables	1950–2000	-	1 km
SWBD^d^	Water bodies	2000	-	30 m
Köppen-Geiger^e^	Climate zones	1976–2000	-	50 km
ISRIC^f^	pH in water	-	-	10 km
Atlas of the Biosphere^g^	Soil moisture	1950–1999	-	50 km
WorldPop^h^	Grid population	2015	-	1 km
SEDAC^i^	HII^m^	1995–2004	-	1 km
SEDAC^i^	Urban extents	1990–2000	-	1 km
SEDAC^i^	IMR^n^	2000	-	4 km
GADM^j^	Geographic administrative boundaries	2012	-	-

^a^All data were accessed on January 1, 2019.

^b^Moderate Resolution Imaging Spectroradiometer (MODIS)/Terra; available at: http://modis.gsfc.nasa.gov/.

^c^Available at: http://www.worldclim.org/current.

^d^Shuttle Radar Topography Mission Water Body Data (SWBD); available at: http://gis.ess.washington.edu/data/vector/worldshore/index.html.

^e^World Maps of Köppen-Geiger climate classification; available at: http://koeppen-geiger.vu-wien.ac.at/shifts.htm.

^f^International Soil Reference and Information Center; available at: https://www.isric.org/.

^g^Available at: http://nelson.wisc.edu/sage/data-and-models/atlas/data.php?incdataset=Soil%20Moisture.

^h^The WorldPop project; available at: http://www.worldpop.org.uk/.

^i^Socioeconomic Data and Applications Center; available at: http://sedac.ciesin.org/.

^j^Global Administrative Areas database; available at: http://www.gadm.org/.

^k^Land surface temperature (LST) day and night.

^l^Normalized difference vegetation index.

^m^Human influence index.

^n^Infant Mortality Rate.

Socioeconomic data such as human influence index (HII), urban extents, and infant mortality rate (IMR) were downloaded from the Socioeconomic Data and Applications Center (Table 1). Geo-referenced water, sanitation, and hygiene (WASH) data for Bangladesh, Nepal, and Pakistan were extracted from the Demographic and Health Surveys (DHS). For India, WASH information were obtained from the Census of India 2011, which were aggregated at administrative division of level three, stratified by rural and urban areas. The following indicators were extracted: proportion of households practicing open defecation, proportion of households with improved sanitation, and proportion of households with improved drinking water sources. An overview of WASH sources and data summaries of the relevant indicators are given in Table 2.

**Table 2:** Overview of WASH sources and data summaries of the relevant indicators by country.

Country			Bangladesh	India	Nepal	Pakistan
Sources			DHS^a^	Census of India^b^	DHS^a^	DHS^a^
Data period			1999–2011	2011	2001–2011	2006
Number of locations			1,661	2,172	800	957
Type of locations			Point	Aggregated at administrative division of level three	Point	Point
Mean proportion (%)	Urban	Sanitation^c^	56.8	69.7	43.2	75.5
		Water^d^	99.4	76.7	92.9	95.1
		Defecation^e^	2.3	25.2	19.9	4.7
	Rural	Sanitation^c^	40.3	31.3	24.1	34.9
		Water^d^	97.8	63.8	80.4	86.3
		Defecation^e^	11.7	63.4	58.3	42.7

^a^Demographic and Health Surveys (DHS); available at: http://dhsprogram.com/.

^b^Census of India 2011; available at: http://censusindia.gov.in/.

^c^Proportion of households with improved sanitation.

^d^Proportion of households with improved drinking water sources.

^e^Proportion of households practicing open defecation.

Visual Fortran version 6.0 (Digital Equipment Corporation; Maynard, United States of America) was employed to extract the environmental and socioeconomic data at survey locations. We linked the survey locations with missing data to the values at the nearest pixels. Surveys aggregated over districts were linked with the average values of the covariates within the districts and were georeferenced using the corresponding centroids.

### Statistical analysis

Survey years were grouped into three periods (before 1980, 1980 to 1999, and from 2000 onwards) to study temporal trends. Continuous variables were standardized to mean zero and standard deviation (SD) one. Based on exploratory analysis, we converted continuous variables into categorical variables based on plotting of disease prevalence with each continuous variable to capture the non-linear relationships. Pearson’s correlation was used to check for continuous variables with a high correlation coefficient (>0.8) to avoid collinearity, while Cramér’s V was applied for categorical variables.

Bayesian variable selection was applied to identify the best set of predictors using a stochastic search approach [23]. For each continuous covariate, a binary indicator was included in the model to indicate the exclusion/inclusion probability of the corresponding covariate. The priors for the coefficients of the covariates were constructed by a narrow spike (i.e., a normal distribution with variance close to zero to shrink the coefficient to zero) and a wide slab (i.e., a normal distribution that supports a non-zero coefficient). Inverse gamma prior distributions were employed for the variance parameters. We selected the covariates with inclusion probabilities (mean posterior distribution of indicators) greater than 0.5 for the final geostatistical analysis. Moreover, an adapted version of the above priors was utilized for categorical variables to include or exclude all categories of the variables simultaneously [24]. An additional indicator was introduced for each continuous variable to select either its linear or non-linear form, as detailed elsewhere [15]. The following 23 variables were considered for Bayesian variable selection: mean diurnal range, isothermality, temperature annual range, annual precipitation, precipitation of driest month, precipitation seasonality, precipitation of warmest quarter, precipitation of coldest quarter, elevation, HII, IMR, LST in the daytime, soil moisture, soil pH, NDVI, distance to the nearest freshwater body, proportion households with improved sanitation, proportion of households with improved water sources, proportion of households with open defecation, survey type (school- or community-based), urban extents, land cover, and climatic zones.

For each soil-transmitted helminth species, Bayesian geostatistical logistic regression models with spatially structured random effects were developed to obtain the spatially explicit estimates of infection risk [25]. Similar models were fitted on WASH indicators for Bangladesh, Nepal, and Pakistan using urban/rural as a covariate, as survey locations of these data were not aligned in space with infection prevalence data. Geostatistical model predictions estimated the WASH indicators at the disease survey locations. Markov chain Monte Carlo (MCMC) simulation was applied to estimate the model parameters in Winbugs version 1.4 (Imperial College London and Medical Research Council; London, United Kingdom) [26]. Two chains were run and convergence was assessed by the Brooks-Gelman-Rubin diagnostic [27].

The model was fitted on a random subset of 80% of the survey locations, and it was validated on the remaining 20% by comparing the observed and predicted prevalence values using the mean predictive error, the area under the curve (AUC) obtained from the receiver-operating characteristic (ROC) curve [28], and the percentages of observations included in the Bayesian credible intervals (BCI) of various probability coverage rates of the predictive distributions [19]. Of note, an AUC between 0.5 and 0.7 indicates a poor discriminative capacity; 0.7–0.9 indicates a reasonable capacity; and >0.9 indicates a very good capacity [28]. A 5 × 5 km grid was overlaid to the study region, resulting in 222,555 pixels. Prediction of infection risk for each soil-transmitted helminth species was done at the centroids of the grid’s pixels using Bayesian kriging [29]. We assumed independence of either species of soil-transmitted helminth and estimated the prevalence of infection by any species using the formula *p*_*S*_ = *p*_*A*_ + *p*_*T*_ + *p*_*h*_ − *p*_*A*_ × *p*_*T*_ − *p*_*A*_ × *p*_*h*_ − *p*_*T*_ × *p*_*h*_ + *p*_*A*_ × *p*_*T*_ × *p*_*h*_, where *p*_*S*_, *p*_*A*_, *p*_*T*_, and *p*_*h*_ indicate the predicted prevalence of any soil-transmitted helminth, *A*. *lumbricoides*, *T*. *trichiura*, and hookworm infections, respectively. To assess the performance of this method, we calculated the mean predictive error, the AUC of the ROC curve, and the percentage of observations included in 95% BCI of the predictive distributions, based on the predicted and the observed overall prevalence.

Population-adjusted prevalence of soil-transmitted helminth infection for each country was estimated by overlaying the pixel-based infection risk on gridded population to obtain the number of infected individuals at each pixel, which was then summed up within country and divided by the country population. The numbers of anthelmintic doses and the numbers of people requiring preventive chemotherapy were estimated at the pixel level according to WHO control guidelines [11], summarized by country. We calculated the annualized pixel-level numbers of anthelmintic doses for school-age children and for pre-school-age children as zero at pixels with estimated prevalence <20%, as the corresponding population at pixels with estimated prevalence ≥20% and <50%, and as double the corresponding population at pixels with estimated prevalence ≥50%. The pixel-level numbers of school-age children and pre-school-age children requiring preventive chemotherapy were calculated as zero at pixels with estimated prevalence <20%, and as the corresponding population at pixels with estimated prevalence ≥20%.

Surveys aggregated over districts were treated as point-level data georeferenced at district centroids. This approach may bias the estimates of the spatial parameters, as it ignores the within-district variation. To assess sensitivity of inferences on the incorporation of the district-level aggregated data into the analysis, we carried out additional analysis by geo-referencing the district-level data to the population-weighted centroids of the corresponding districts. Results of parameter estimates, population-adjusted predicted prevalence and high-resolution risk maps were compared between the two approaches.

## Results

### Data summaries

**Fig 1:**
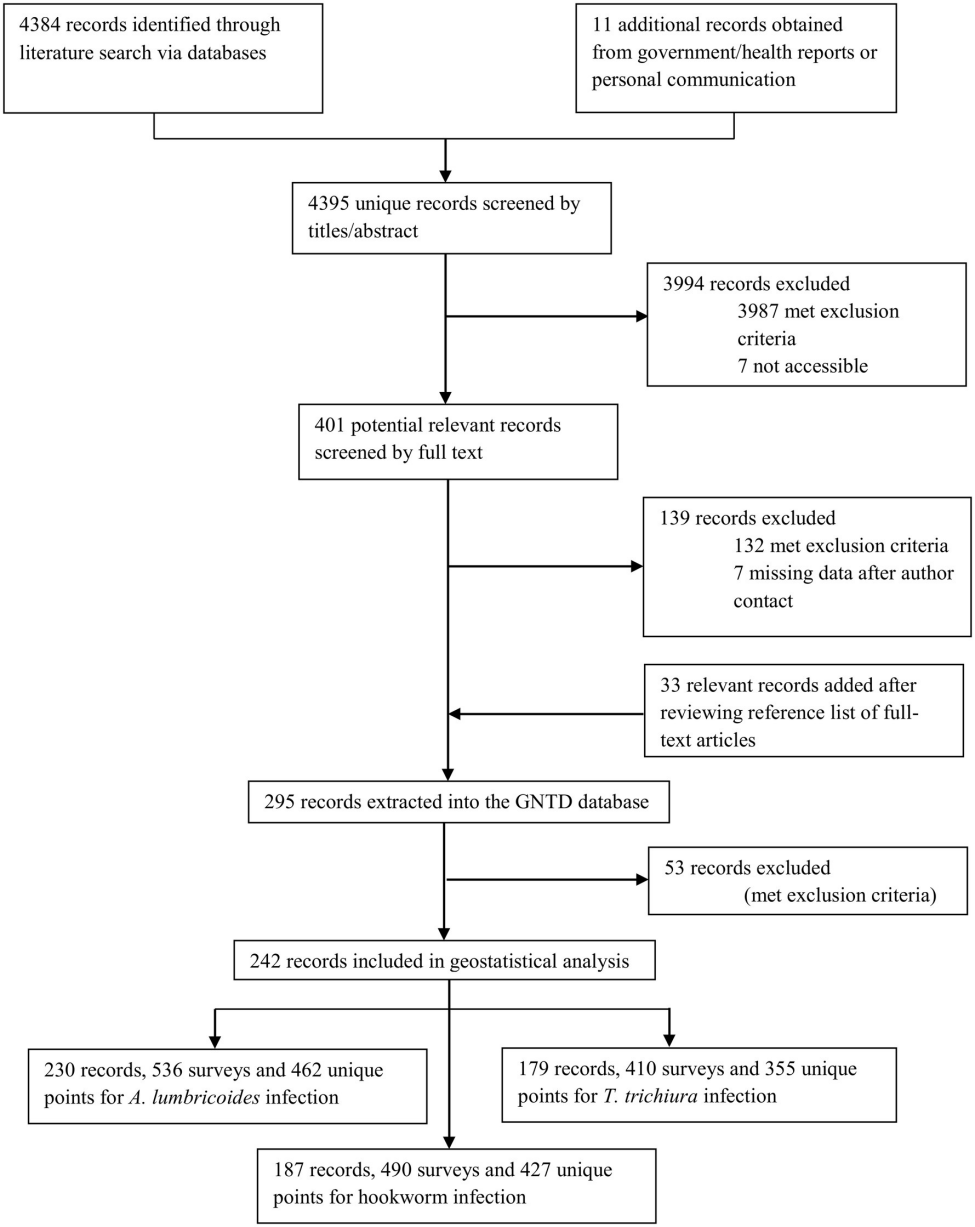
Data selection flow chart.

We identified 4,384 records by systematically reviewing the peer-reviewed literature, while an additional 11 records stemmed from the grey literature and personal communication for the four mainland countries of Bangladesh, India, Nepal, and Pakistan. After excluding records according to our study protocol, 242 records remained, resulting in 536 surveys for *A*. *lumbricoides* at 462 unique locations, 410 surveys for *T*. *trichiura* at 355 unique locations, and 490 surveys for hookworm at 427 unique locations (Fig 1). Only 24 surveys reported overall prevalence of soil-transmitted helminth infection.

**Fig 2:**
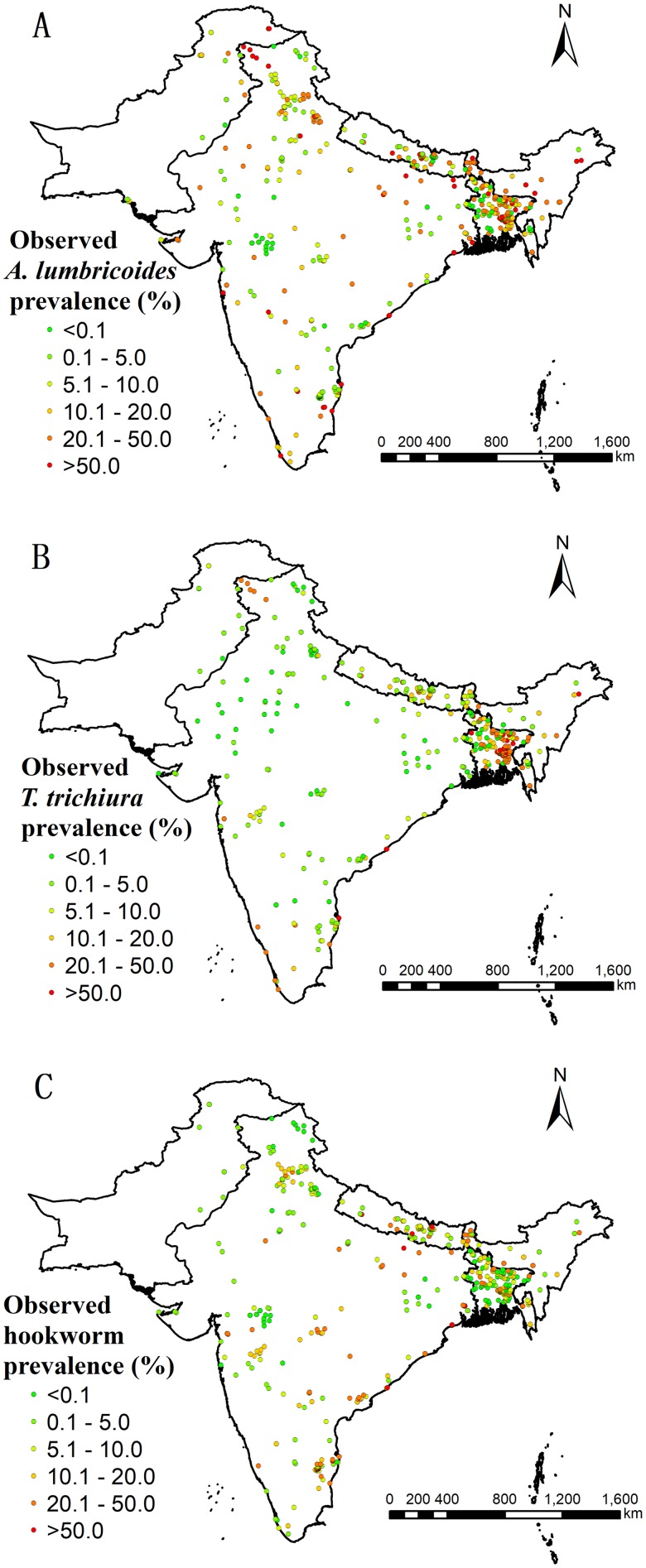
Survey locations and observed prevalence over the study region. (A) *A*. *lumbricoides*, (B) *T*. *trichiura*, and (C) hookworm.

Table 3 shows an overview of the soil-transmitted helminth surveys included in the final analysis, stratified by country. Fig 2 displays the geographic distribution of locations and observed prevalence for each soil-transmitted helminth species. Supporting Information S1 Fig shows the distribution of survey years, categorized by different periods (before 1980, 1980 to 1999, and from 2000 onwards). There were only few surveys in the southern and western parts of Pakistan and in the central part of India. A summary of diagnostic methods of surveys are shown in Supporting Information S1 Table. Search results for the remaining countries of South Asia (i.e., Afghanistan, Bhutan, the Maldives, and Sri Lanka) are listed in Supporting Information S2 Table.

**Table 3:** Overview of soil-transmitted helminth infection surveys in four countries of South Asia.

Country		Bangladesh	India	Nepal	Pakistan	Total
		***A*. *lumbricoides***				
Relevant papers		27	136	49	18	230
Total surveys/locations		134/119	301/264	75/55	26/24	536/462
Survey type (surveys/locations)	School	9/6	99/92	46/36	12/11	166/145
	Community	125/113	202/172	29/19	14/13	370/317
Location type (surveys/locations)	Point	107/102	156/135	36/26	12/11	311/274
	District	27/17	145/129	39/29	14/13	225/188
Period		1957–2012	1963–2017	1995–2015	1976–2015	1957–2017
Year of survey (surveys/locations)	<1980	8/6	43/38	0/0	6/6	57/50
	1980–1999	18/9	82/70	14/10	10/10	124/99
	≥2000	108/107	176/163	61/49	10/9	355/328
Raw prevalence (%)		55.0	17.3	17.7	13.1	19.7
		***T*. *trichiura***				
Relevant papers		23	101	44	11	179
Total surveys/locations		129/117	199/174	69/52	13/12	410/355
Survey type (surveys/locations)	School	8/5	94/87	43/34	5/5	150/131
	Community	121/112	105/87	26/18	8/7	260/224
Location type (surveys/locations)	Point	107/102	103/88	35/26	3/3	248/219
	District	22/15	96/86	34/26	10/9	162/136
Period		1957–2012	1963–2014	1995–2015	1976–2014	1957–2015
Year of survey (surveys/locations)	<1980	7/5	31/26	0/0	2/2	40/33
	1980–1999	14/8	53/42	12/10	4/4	83/64
	≥2000	108/107	115/110	57/45	7/6	287/268
Raw prevalence (%)		44.1	6.6	14.5	2.8	10.3
		**Hookworm**				
Relevant papers		21	114	42	10	187
Total surveys/locations		129/117	284/248	65/51	12/11	490/427
Survey type (surveys/locations)	School	7/5	81/73	41/33	4/4	133/115
	Community	122/112	203/175	24/18	8/7	357/312
Location type (surveys/locations)	Point	107/105	172/150	33/26	2/2	314/283
	District	22/12	112/98	32/25	10/9	176/144
Period		1957–2012	1962–2017	1995–2015	1978–2012	1957–2017
Year of survey (surveys/locations)	<1980	7/6	52/43	0/0	1/1	60/50
	1980–1999	18/9	78/67	10/8	5/5	111/89
	≥2000	104/104	154/143	55/46	6/5	319/298
Raw prevalence (%)		13.2	17.4	15.7	3.4	16.2

### Variable selection and geostatistical modeling

**Fig 3:**
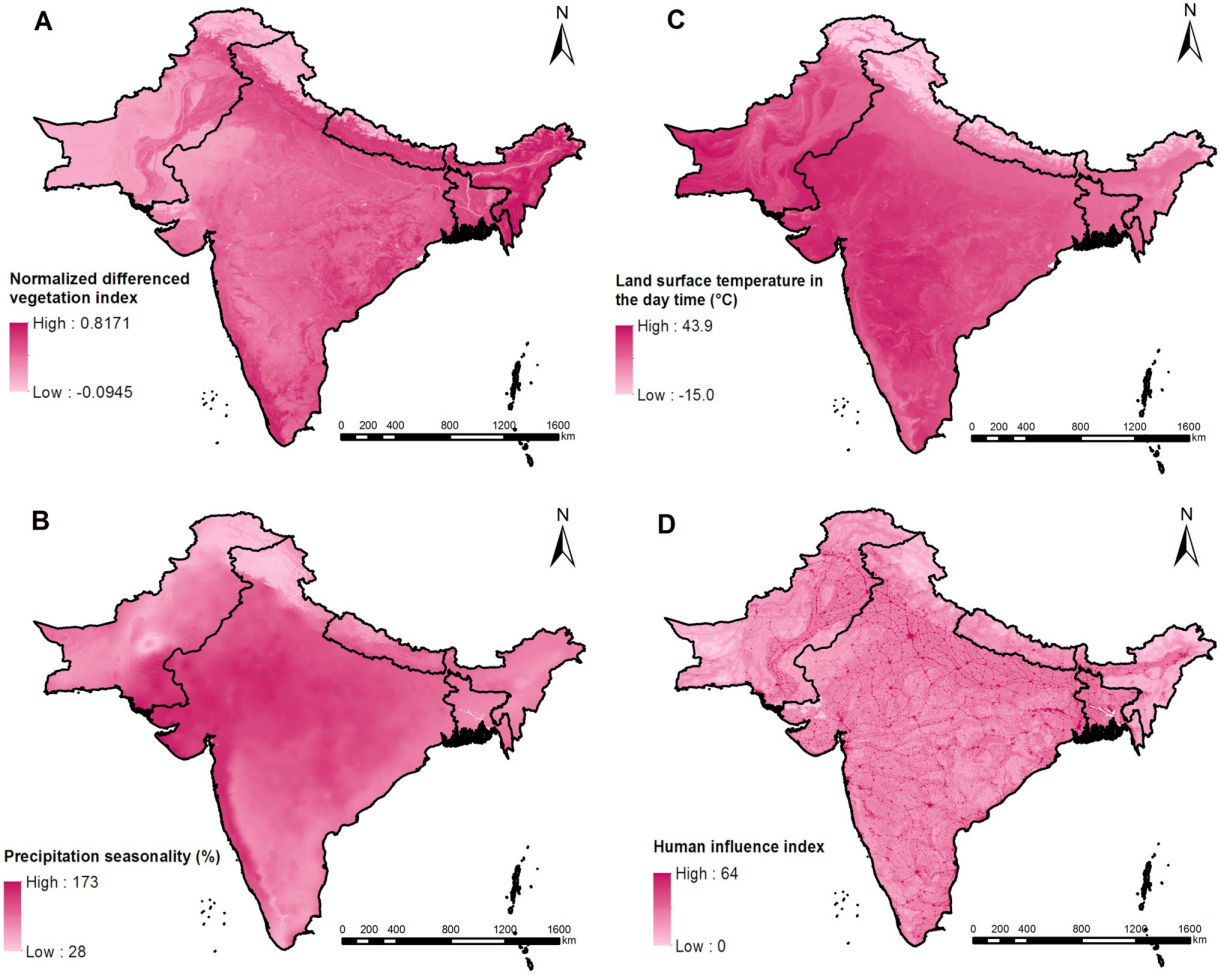
Spatial distributions of the selected variables. (A) Normalized difference vegetation index, (B) precipitation seasonality, (C) land surface temperature in the day time, and (D) human influence index.

**Fig 4:**
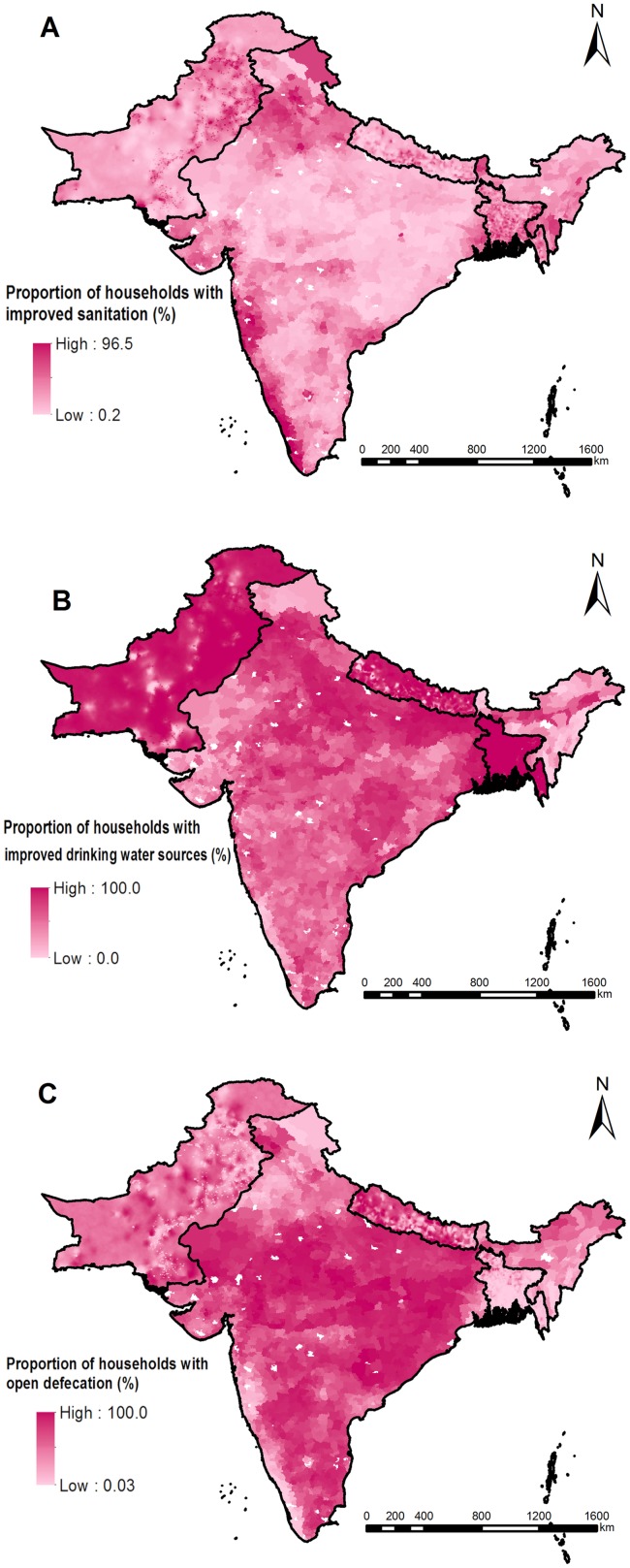
Spatial distribution of the WASH indicators. (A) Proportion of households with improved sanitation, (B) proportion of households with improved water sources, and (C) proportion of households with open defecation.

The selected variables from Bayesian variable selection are listed in Table 4. Maps of spatial distributions of the selected variables and the WASH indicators are shown in Figs 3 and 4. In the final geostatistical logistic regression models, the infection risk decreased from 2000 onwards for hookworm, while the infection risk first increased in 1980–1999 and then decreased from 2000 onwards for *A*. *lumbricoides* and *T*. *trichiura* (Table 4). A negative association was identified for the prevalence of *A*. *lumbricoides* with LST in the daytime, whereas a positive association was found with HII. There was no significant difference between prevalence of *A*. *lumbricoides* in school-age children and that in the community population. Negative associations were identified for *T*. *trichiura* infection risk with LST in the daytime and precipitation seasonality. Positive associations was found for hookworm infection risk with proportion of households with open defecation and average NDVI.

**Table 4:** Posterior summaries (median and 95% Bayesian credible interval) of the geostatistical model parameters.

***A*. *lumbricoides***	**Estimate**
Period (<1980) ^a^	
1980–1999	0.61 (0.52; 0.72)^b^
≥2000	-0.00 (-0.10; 0.11)
Survey type (school-based)^a^	
Community-based	-0.03 (-0.11; 0.05)
Land surface temperature in the day time (25–30°C)^a^	
≤8	0.04 (-4.18; 3.66)
8–20	1.49 (0.79; 2.29)^b^
20–25	0.87 (0.31; 1.53)^b^
30–35	-0.50(-0.10; -0.08)^b^
>35	-0.74 (-1.38; 0.13)
Human influence index (≤22)^a^	
22–32	0.13 (-0.40; 0.63)
>32	0.80 (0.31; 1.48)^b^
Range (km)	109.0 (63.9; 174.0)
Spatial variance (σ^2^_sp_)	2.18 (1.56; 3.05)
Non-spatial variance (σ^2^_nonsp_)	1.07 (0.74; 1.53)
***T*. *trichiura***	**Estimate**
Period (<1980)^a^	
1980–1999	1.23 (1.09; 1.37)^b^
≥2000	0.32 (0.18; 0.46)^b^
Precipitation seasonality (90–110%)^a^	
≤70	-0.59 (-1.74; 0.61)
70–90	0.55 (-0.03; 1.18)
110–130	-0.96 (-1.64; -0.42)^b^
>130	-1.86 (-2.80; -0.84)^b^
Land surface temperature in the day time (≤26.5°C)^a^	
26.5–31.0	-0.30 (-0.93; 0.47)
>31.0	-1.50 (-2.21; -0.35)^b^
Range (km)	134.0 (65.0; 240.9)
Spatial variance (σ^2^_sp_)	1.99 (1.23; 3.00)
Non-spatial variance (σ^2^_nonsp_)	1.03 (0.58; 1.74)
**Hookworm**	**Estimate**
Period (<1980)^a^	
1980–1999	-0.60 (-0.77; -0.40)^b^
≥2000	-0.65 (-0.83; -0.43)^b^
Normalized difference vegetation index (≤0.40)^a^	
0.40–0.53	0.31 (-0.42; 0.77)
>0.53	0.70 (0.07; 1.33)^b^
Open defecation (≤15%)^a^	
15–60	0.61 (0.08; 1.17)^b^
>60	0.15 (-0.52; 0.67)
Range (km)	152.6 (71.6; 286.9)
Spatial variance (σ^2^_sp_)	1.89 (1.20; 3.00)
Non-spatial variance (σ^2^_nonsp_)	1.15 (0.82; 1.54)

^a^In brackets, baseline values are reported;

^b^important effect based on 95% Bayesian credible interval (BCI).

### Model validation

Model validation indicated that the geostatistical logistic regression models were able to correctly estimate (within the 95% BCI) 84.1%, 80.6%, and 74.4% of locations for *A*. *lumbricoides*, hookworm, and *T*. *trichiura*, respectively. The mean errors for hookworm, *A*. *lumbricoides*, and *T*. *trichiura* were 4.9%, 5.0%, and 5.7%, respectively, suggesting our models may under-estimate the infection risk of the three soil-transmitted helminth species. The AUCs for *A*. *lumbricoides*, *T*. *trichiura*, and hookworm were 0.80, 0.79, and 0.70, respectively, indicating a good overall predictive performance. With regard to the overall prevalence, the 95% BCI coverage, the mean error, and the AUC were 100%, 9.7%, and 0.88, respectively.

### Predictive risk maps

**Fig 5:**
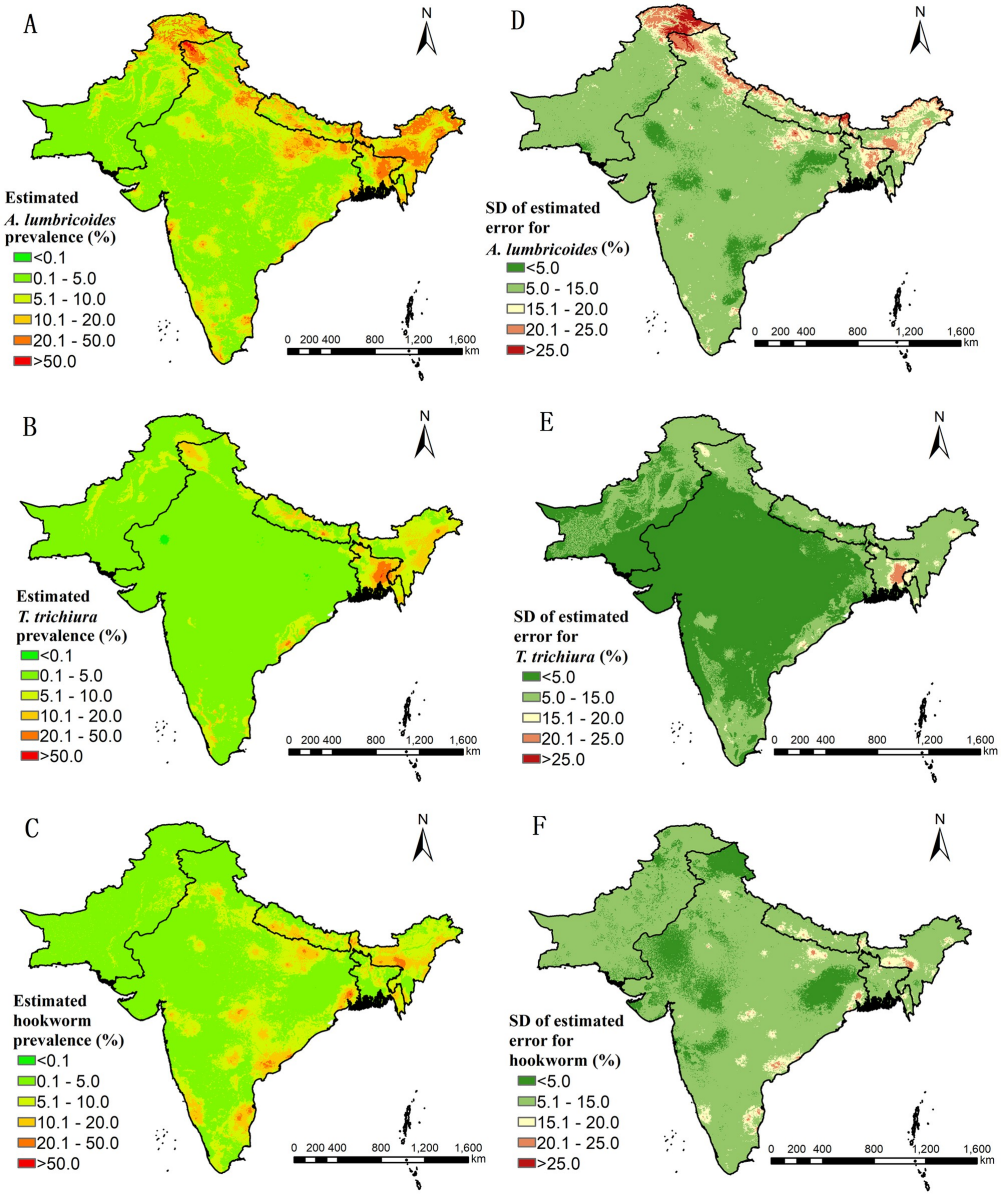
Species-specific model-based predictive risk maps from 2000 onwards. Predictive prevalence based on the median of the posterior predictive distribution of infection risk for (A) *A*. *lumbricoides*, (B) *T*. *trichiura*, and (C) hookworm. Prediction uncertainty based on the standard deviation of the posterior predictive distribution of infection risk for (D) *A*. *lumbricoides*, (E) *T*. *trichiura*, and (F) hookworm.

**Fig 6:**
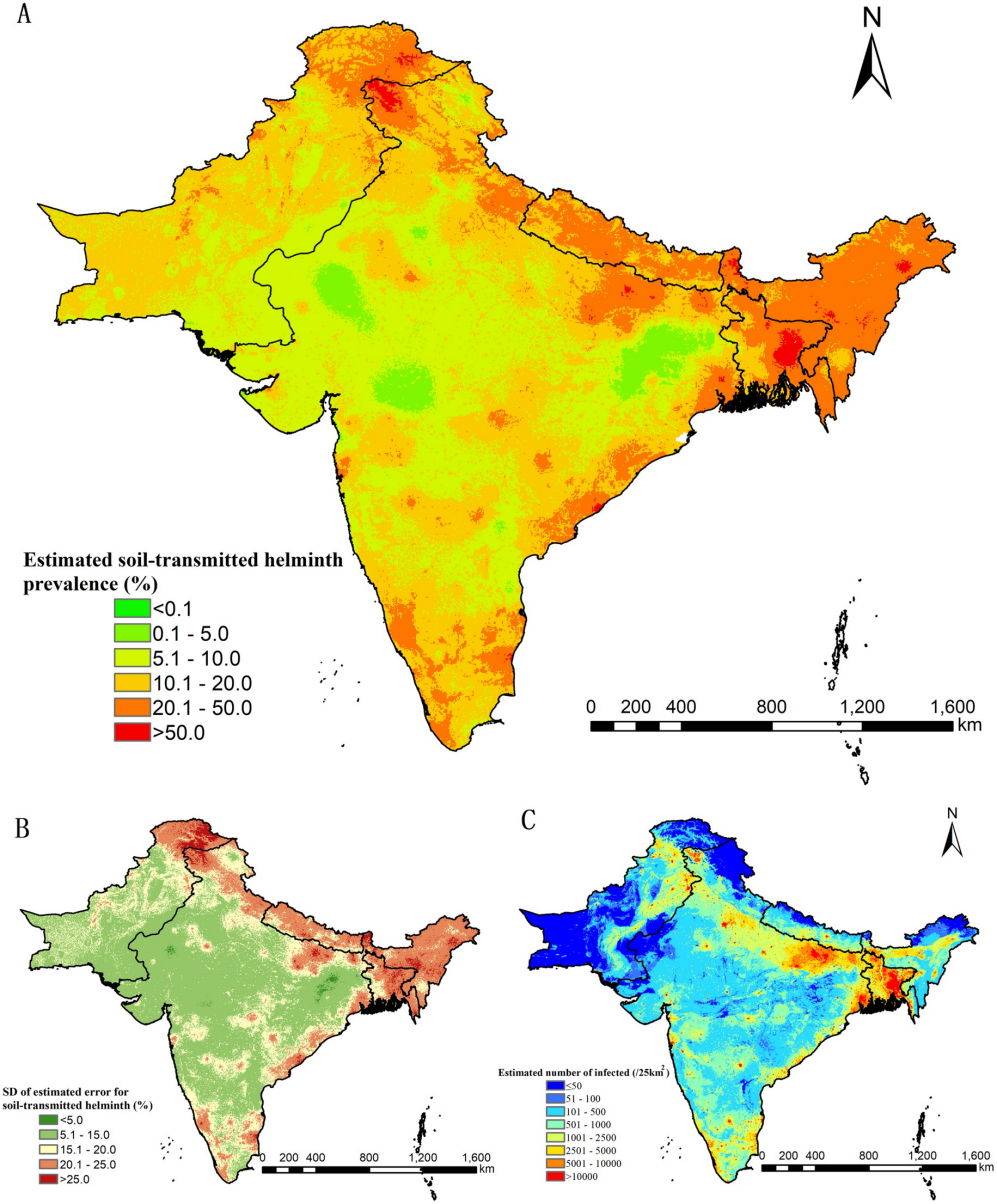
Model-based predictive risk map of any soil-transmitted helminth infection from 2000 onwards. (A) Predictive prevalence based on the median of the posterior predictive distribution of infection risk. (B) Prediction uncertainty based on the standard deviation of the posterior predictive distribution of infection risk. (C) Number of infected people based on the predictive prevalence and gridded population of 2015.

Fig 5A–5C and 5D–5F present the species-specific predictive risk maps and the corresponding prediction uncertainty, respectively. A predictive infection risk map of any soil-transmitted helminth infection and a map of the corresponding prediction error are shown in Fig 6A and 6B. Moderate to high prevalence (>20%) of *A*. *lumbricoides* was mainly predicted in eastern parts of Bangladesh and some northern parts of Pakistan and India. Low prevalence (<5%) was predicted in areas of southern Pakistan and central India. Most of the study region had low prevalence (<5%) of *T*. *trichiura* infection, while the eastern areas of Bangladesh were characterized by moderate to high prevalence (>20%). Moderate to high hookworm prevalence (>20%) was predicted in some areas of southern and eastern India.

### Estimates of population-adjusted prevalence and number of people infected

Table 5 summarizes the population-adjusted predicted prevalence and estimated number of individuals infected with soil-transmitted helminths, stratified by country. Fig 6C shows the estimated number of individuals infected with any soil-transmitted helminth in South Asia. In the whole study region, the overall population-adjusted predicted prevalence of *A*. *lumbricoides*, *T*. *trichiura*, and hookworm were 12.6% (95% BCI: 10.8–14.8%), 4.9% (95% BCI: 4.2–6.0%), and 8.4% (95% BCI: 6.9–10.0%), respectively, corresponding to 206 million (95% BCI: 177–242 million), 80 million (95% BCI: 69–98 million), and 139 million (95% BCI: 114–164 million) infected individuals. The overall population-adjusted predicted prevalence of infected with any soil-transmitted helminth infection was 22.1% (95% BCI: 20.2–24.1%), which is equivalent to 361 million (95% BCI: 330–395 million) infected individuals. The annual treatment needs for school-age children requiring preventive chemotherapy with albendazole or mebendazole according to WHO’s guidelines was estimated at 165 million doses (95% BCI:146–185 million). Of note, we estimated that approximately one fourth of infected people were concentrated in low-risk areas (i.e., settings with predicted prevalence below the WHO preventive chemotherapy threshold 20%), which accounts for approximately 87 million (95% BCI: 81–94 million) infected people or 17 million (95% BCI: 16–19 million) infected school-age children who are not being targeted by preventive chemotherapy, strictly following WHO treatment strategies (Supporting Information S3 Table).

**Table 5:** Population-adjusted predicted prevalence (%) and number of individuals (×10^6^) infected with soil-transmitted helminths, stratified by country^a^.

Country		Bangladesh	India	Nepal	Pakistan	Total
Population		157.35	1,258.49	32.67	188.82	1,637.33
Population of school-age children		31.35	243.54	7.46	41.45	323.80
Population of pre-school-age children		11.99	95.00	2.57	19.72	129.28
*A*. *lumbricoides*	Prevalence	20.8 (17.4; 24.5)	11.7 (9.9; 13.9)	14.1 (11.1; 18.4)	11.1 (8.5; 14.5)	12.6 (10.8; 14.8)
	No. of entire population infected	32.79 (27.44; 38.47)	147.56 (124.86; 175.35)	4.61 (3.63; 6.03)	20.94 (16.11; 27.46)	206.23 (176.58; 241.83)
	No. of school-age children infected	6.63 (5.52; 7.72)	28.90 (24.28; 34.71)	1.08 (0.84; 1.38)	4.69 (3.70; 6.25)	41.45 (35.58; 48.49)
*T*. *trichiura*	Prevalence	19.2 (16.2; 22.6)	3.3 (2.7; 4.2)	7.2 (5.1; 9.9)	3.6 (2.4; 5.5)	4.9 (4.2; 6.0)
	No. of entire population infected	30.26 (25.46; 35.55)	41.02 (33.39; 52.67)	2.35 (1.67; 3.23)	6.77 (4.53; 10.42)	80.38 (68.55; 97.79)
	No. of school-age children infected	6.03 (5.07; 7.08)	7.94 (6.46; 10.19)	0.54 (0.38; 0.74)	1.49 (0.99; 2.29)	15.99 (13.66; 19.47)
Hookworm	Prevalence	9.4 (7.3; 11.9)	8.7 (6.9; 10.5)	10.8 (8.0; 14.7)	6.0 (4.1; 8.7)	8.4 (6.9; 10.0)
	No. of entire population infected	14.71 (11.48; 18.79)	108.90 (86.72; 131.68)	3.53 (2.62; 4.81)	11.32 (7.80; 16.37)	138.52 (113.62; 163.69)
	No. of school-age children infected	2.93 (2.29; 3.74)	21.07 (16.78; 25.48)	0.81 (0.6; 1.10)	2.48 (1.71; 3.59)	27.32 (22.40; 32.21)
Any soil-transmitted helminth	Prevalence	37.8 (34.6; 41.3)	20.5 (18.4; 22.5)	27.1 (23.4; 30.6)	18.4 (15.9; 22.6)	22.1 (20.2; 24.1)
	No. of entire population infected	59.43 (54.37; 64.98)	257.56 (231.78; 283.51)	8.86 (7.65; 10.01)	34.69 (30.05; 42.70)	361.04 (330.53; 395.06)
	No. of school-age children infected	11.92 (10.92; 13.07)	50.17 (45.33; 55.38)	2.04 (1.76; 2.29)	7.71 (6.58; 9.51)	71.99 (66.14; 79.08)
School-age children requiring preventive chemotherapy (×10^6^)	Model-based estimate	30.68 (28.03; 33.76)	112.67 (97.47; 128.30)	5.02 (4.09; 5.88)	16.42 (12.86; 21.92)	164.73 (146.32; 185.34)
	WHO estimate^b^	31.28	159.19	5.45	21.76	217.69
Pre-school-age children requiring preventive chemotherapy (×10^6^)	Model-based estimate	11.73 (10.72; 12.91)	43.95 (38.02; 50.05)	1.73 (1.41; 2.03)	7.81 (6.12; 10.43)	65.19 (57.91; 73.64)
	WHO estimate^b^	16.20	63.87	2.19	9.29	91.56
Number of anthelmintic doses for school-age children (×10^6^)		30.68 (28.03; 33.76)	112.67 (97.47; 128.3)	5.02 (4.09; 5.88)	16.42 (12.86; 21.92)	164.73 (146.32; 185.34)
Number of anthelmintic doses for pre-school-age children (×10^6^)		11.73 (10.72; 12.91)	43.95 (38.02; 50.05)	1.73 (1.41; 2.03)	7.81 (6.12; 10.43)	65.19 (57.91; 73.64)

^a^Estimates were based on gridded population of 2015; calculations were based on the median and 95% BIC of the posterior predictive distribution of the infection risk from 2000 onwards;

^b^Obtained from WHO, PCT databank (http://www.who.int/neglected_diseases/preventive_chemotherapy/sth/en/) for the year 2015.

Bangladesh showed the highest population-adjusted predicted prevalence of *A*. *lumbricoides* (20.8%; 95% BCI: 17.4–24.5%), *T*. *trichiura* (19.2%; 95% BCI: 16.2–22.6%), and any soil-transmitted helminth species (37.8%; 95% BCI: 34.6–41.3%). Nepal had the highest predicted prevalence of hookworm infection (10.8%; 95% BCI: 8.0–14.7%) and the second highest of any soil-transmitted helminth infection in the region. India had the largest numbers of individuals estimated to be infected with *A*. *lumbricoides* (148 million; 95% BCI: 125–175 million), *T*. *trichiura* (41 million; 95% BCI: 33–53 million), hookworm (109 million; 95% BCI: 87–132 million), and any soil-transmitted helminth infection (258 million; 95% BCI: 232–284 million).

## Discussion

We pursued a systematic review to collect available georeferenced data pertaining to prevalence of soil-transmitted helminth infections in South Asia, using rigorous Bayesian variable selection to identified important predictors, and developed Bayesian geostatistical logistic regression models for spatially explicit estimates of infection risk. To our knowledge, we present the first model-based, high-resolution infection risk estimates of the three main soil-transmitted helminth species as well as a risk map of any soil-transmitted helminth infection in South Asia. The latter map is particularly relevant in terms of disease control as preventive chemotherapy with albendazole or mebendazole is based on the overall prevalence of any soil-transmitted helminth, usually estimated for the school-age population [30,31].

Our estimates suggest that, in 2015, approximately 12.6% (95% BCI: 10.8–14.8%), 4.9% (95% BCI: 4.2–6.0%), and 8.4% (95% BCI: 6.9–10.0%) of the population in South Asia were infected with *A*. *lumbricoides*, *T*. *trichiura*, and hookworm, respectively, corresponding to population estimates of 206 million (95% BCI: 177–242 million), 80 million (95% BCI: 69–98 million), and 139 million (95% BCI: 114–164 million) for the three species, respectively. We estimated lower numbers of infection for *A*. *lumbricoides* and *T*. *trichiura*, while similar numbers of infection for hookworm, compared to previous estimates in 2010, put forth by Pullan and colleagues [3]. Of note, the later estimates were obtained by direct empiric approaches based on aggregated prevalence data at administrative level two or higher [3], while our risk predictions were based on rigorous Bayesian geostatistical models that allow our aggregated estimates to be geographically weighted, thus taking into account the heterogeneous distributions of disease risk and population at risk within the studied countries. We estimated that the number of school-age children requiring preventive chemotherapy was 165 million (95% BCI: 146–185 million), which is lower than the 218 million estimated by WHO in 2015 [13]. The latter was based on an algorithm taking into account the availability of data in the country’s national plan of action, epidemiologic information, ecologic situation, and sanitation [32], while we estimated the numbers through high-resolution, model-based risk profiles based on all available geo-referenced survey data and important environmental and socioeconomic information. Besides, we provided estimates of the number of anthelmintic doses (165 million, 95% BCI: 146–185 million), which is especially important for financial planning. One cannot tell how many drugs are needed when only the number of population requiring preventive chemotherapy is available, as the treatment frequency (i.e., once or twice per year) is unknown. By considering costs of US$ 0.03 for albendazole per treatment [33,34], the annual drug cost for preventive chemotherapy for school-age children in South Asia was estimated to be US$ 4.9 million (95% BCI: 4.4–5.6 million). These estimates are useful for decision makers and funding agencies.

Our final models had reasonable predictive ability, as revealed by model validation suggesting that they were able to correctly predict 84.1%, 80.6%, and 74.4% of locations for *A*. *lumbricoides*, hookworm, and *T*. *trichiura*, respectively. However, our models may under-estimate the true species-specific prevalence of each soil-transmitted helminth species, as the mean errors, which show the overall tendency of prediction bias, were larger than zero for all three species. This bias may result from the distribution of survey locations, the data characteristics, and the model assumptions. We estimated an overall prevalence of any soil-transmitted helminth infection by assuming independence of the three species, which might over-estimate the reported prevalence, as some researchers suggested a positive association between *A*. *lumbricoides* and *T*. *trichiura* [35,36]. To assess the model performance for overall soil-transmitted helminth prevalence, we compared model-based predictions with the observed prevalence at the 24 survey locations reporting overall prevalence. The positive mean error indicated that our model may under-estimate the true prevalence. However, all observed prevalence values fell within the 95% BCI of predicted prevalence and the AUC was close to 0.9, showing a good model performance.

On the other hand, our compiled survey data must be treated with caution, as sampling effort and diagnostic approaches were not uniform. For example, more than 25% of the surveys employed the widely used Kato-Katz technique, while more than 70% had missing information on the sampling effort (e.g., number of stool samples and total number of slides analyzed per sample). However, the diagnostic sensitivity relies on sampling effort as well as on the infection intensity [37]. In the absence of sufficient information and to avoid introducing debatable assumptions, we did not consider the diagnostic error and therefore our predictions might under-estimate the true prevalence [37,38]. However, our results still provide reliable information as, in most cases, warranting preventive chemotherapy is based on diagnostic prevalence rather than true prevalence. To avoid selection bias, we excluded studies involving specific groups that might not be representative. The final survey data for analysis included both community- and school-based studies. Survey type (community- or school-based) was included as a potential predictor in the variable selection procedure and the final geostatistical models adjusted for its effect on the disease risk (in case it was selected). We did not adjust for the age and gender distribution in each study. This information, anyways, was not available for most studies, and hence, it is difficult to appreciate this potential source of bias.

We identified several climatic and environmental factors that were associated with soil-transmitted helminth infection, such as LST in the daytime, precipitation seasonality, and NDVI. Our findings are consistent with other reports emphasizing that environmental conditions play an important role in the transmission of helminths [39–41]. A similar relationship was found between LST in the daytime and *T*. *trichiura* infection risk in the People’s Republic of China [15]. Socioeconomic factors impact the transmission of soil-transmitted helminths, mainly via influencing the behavior of people [42]. We found that HII showed a positive association with *A*. *lumbricoides*, indicating that direct human influence on ecosystems may have an effect on helminth transmission. Improvements of WASH are considered as interventions for sustainable control of soil-transmitted helminthiasis [43]. A systematic review and meta-analysis compiling results from individual-level studies showed a significant relationship between WASH and soil-transmitted helminth infection risk [44]. Results from our systematic review suggest that higher proportions of households practicing open defecation had a positive effect on hookworm infection risk, which is consistent with previous observations [45]. However, the Bayesian variable selection did not identify important WASH indicators for either *A*. *lumbricoides* or *T*. *trichiura*. The effect of WASH can differ between genders, or sub-groups with exposure-related behavior patterns. Because we aggregated data within villages or areas, it may have been difficult to detect those variations [19,46,47]. In addition, bias in prediction of the WASH indicators might exist, as each country implemented their own survey with different methodologies and in different years.

To avoid data sparsity, especially in areas without recent surveys, we included into our analysis all data from 1950 onwards and took into account the temporal effects on the disease risk by considering the survey period as a categorical covariate. However, a considerable amount of point-specific survey data could not be accessed; indeed, approximately 40% of our survey data were aggregated at district level, and were not available at survey locations even after contacting the authors. To avoid data scarcity, we treated the data as point-specific georeferenced at the centroids of the district. The mean size of the corresponding districts was around 6500 km^2^. This approach may lead to bias in the estimates of spatial parameters. We did an additional analysis by geo-referencing the district-level data to population-weighted centroids of the corresponding districts. Results related to the parameter estimates, the population-adjusted predicted prevalence, and the high-resolution risk maps (Supporting Information S4 and S5 Tables and S2 Fig, respectively) were quite similar to the former estimates, indicating the reliability of the approach used in our manuscript.

We encourage researchers to share data disaggregated at the survey locations, to support secondary analyses for estimates of disease burden at high spatial resolution. Our study identified areas with sparse data, which can help in the planning of future surveys. Furthermore, national surveys after large-scale deworming are important for monitoring and assessing control interventions and for avoiding overtreatment of populations if the treatment estimates relied on historic data. On the other hand, historic data reflect untreated populations, giving possibly a better indication of transmission intensity and risk of resurgence than more contemporary, post-treatment data. Even though we excluded data from intervention studies or locations where preventive chemotherapy occurred within one year, if such information was mentioned in the corresponding literature, we could not obtain detailed geographic information of large preventive chemotherapy programs in the whole study region. In addition, it is noted that India has implemented mass drug administration for lymphatic filariasis with almost 100% geographical coverage, and Bangladesh and Nepal also did so with high rates of coverage [6]. Hence, we assumed that the effect of preventive chemotherapy for lymphatic filariasis was similar across the study region.

We estimated low-to-moderate (<50%) prevalence of hookworm infection in the northeastern part of Maharashtra State in India. Pullan and Brooker [48] put forth very low risk of hookworm in these areas (prevalence <0.1%). However, their estimates were not supported by observed survey data in several villages of Nagpur district, which shows prevalence of hookworm higher than 15% [49]. On the other hand, our models might over-estimate the risk of soil-transmitted helminth infection in the very high mountainous areas of the northern part of the study region, where the prediction uncertainty was high. Due to lack of data in these areas, further surveys are needed in order to derive more precise estimates. Nevertheless, the predictions of the northern very high mountainous areas did not influence much the population-adjusted predicted prevalence as the population density and the estimated number of infected people in those areas were quite low (Fig 5C). We tried to collect all relevant data through both major search engines and other grey literature, with no restriction of language and date of survey and publication. However, there may be un-reported survey data that we failed to identify. We excluded 14 potential relevant records due to inaccessibility and missing information. We also excluded survey data aggregated over large study regions at country or province-level. We had low geographical coverage of studies in Pakistan where few survey data were available in the southern and western parts of the country. However, the estimates are based on geostatistical models, which get their predictive strength from other areas with large amount of data allowing more accurate estimation of the relation between the disease risk and its predictors. Such models are powerful statistical tools for predicting disease risk in areas with sparse data; yet, risk estimates in regions with low study coverage should be interpreted cautiously.

Our results revealed that the prevalence of any soil-transmitted helminth infection was higher than or close to 20% in all the four South Asian countries subjected to detailed Bayesian-based geostatistical risk profiling, thus more efforts are needed to focus on control and intervention activities in these countries. We found negligible differences between the infection risk in community population and that of school-age children for all three species. These findings support suggestions of other researchers that control strategies focusing on school-based deworming need to be reassessed and extended to other populations (e.g., pre-school-age children, women of reproductive age, and adults at high-risk of occupational exposure) or to the whole community [16,50,51].

We do not provide estimates for Afghanistan, Bhutan, and the island countries of the Maldives and Sri Lanka. In fact, only very sparse georeferenced data were obtained by our systematic review for Afghanistan, Bhutan, and the Maldives, and thus, it was difficult to infer reliable estimates (S2 Table). Even though surveys on soil-transmitted helminth infection were carried out in Bhutan in 1985, 1986, 1989, and 2003, data with precise survey locations were not available [20]. To our knowledge, Bhutan has had a school deworming program in place since 1988, but detailed reports on school deworming are not available [20]. The survey conducted in 2003 observed an overall prevalence of 16.5% for soil-transmitted helminth infection in five schools of the Western region, suggesting a continuation of deworming was needed in the country [20]. There are two available surveys pertaining to the epidemiology of soil-transmitted helminth infections carried out in recent years in Afghanistan. First, a baseline parasitological survey before a nationwide deworming campaign carried out in February and March 2003. Second, an intestinal parasitic infection survey conducted in the eastern part between November 2013 and April 2014 [52,53]. The latter was carried out in one school in Ghazni province, while data of the first were only available at provincial level (administrative division of level one). Both surveys showed moderate to high prevalence (>20%) of soil-transmitted helminth infection and urged effective interventions to control infections in the country. On the other hand, we did not include Sri Lanka for further analysis because data disaggregated at village/school level were not publicly available after 2000. Sri Lanka implemented a major deworming program between 1994 and 2005 and it is considered a country where preventive chemotherapy on soil-transmitted helminth infections is not necessary any longer, according to the observed low prevalence from a national survey conducted in 2003 [12]. However, a school-based cross-sectional survey conducted in 2009 reported that the prevalence bounced back after cessation of preventive chemotherapy to above 20% in four districts of plantation sector (Kandy, Kegalle, Nuwara Eliya, and Ratnapuram), suggesting that effective sustainable control activities should be undertaken in this sector in order to maintain a low prevalence [54].

In conclusion, we present the first model-based, high-resolution risk estimates of soil-transmitted helminth infection in four countries of South Asia, using data obtained from a systematic review and applying rigorous Bayesian geostatistical modeling for prediction based on environmental and socioeconomic predictors. The risk maps provide an estimate of the geographic distribution of the infection and highlight the need for up-to-date surveys to accurately evaluate the disease burden in the region.

## Supporting information

S1 Checklist PRISMA checklist. (DOC)

S1 Table Overview of diagnostic methods of surveys, stratified by country. (DOCX)

S2 Table Overview of soil-transmitted helminth surveys in the remaining countries of South Asia (Afghanistan, Bhutan, the Maldives, and Sri Lanka). (DOCX)

S3 Table Number (×106) and percentage (%) of infected individuals living in areas with low, moderate, and high risk of soil-transmitted helminth infection, stratified by country. (DOCX)

S4 Table Posterior summaries (median and 95% Bayesian credible interval) of the model parameters, using population-weighted centroids as representative locations for district-level survey data. (DOCX)

S5 Table Population-adjusted predicted prevalence (%) and number of individuals (×106) infected with soil-transmitted helminthsa, using population-weighted centroids as representative locations for district-level survey data. (DOCX)

S1 Fig The distribution of survey years categorized by different periods (before 1980, 1980 to 1999, and from 2000 onwards). (A) *A*. *lumbricoides*, (B) *T*. *trichiura*, and (C) hookworm.
(TIF)

S2 Fig Predictive risk maps from 2000 onwards, using population-weighted centroids as representative locations for district-level survey data. Predictive prevalence based on the median of the posterior predictive distribution of infection risk for (A) *A*. *lumbricoides*, (B) *T*. *trichiura*, (C) hookworm, and (D) any soil-transmitted helminth infection. Prediction uncertainty based on the standard deviation of the posterior predictive distribution of infection risk for (E) *A*. *lumbricoides*, (F) *T*. *trichiura*, (G) hookworm, and (H) any soil-transmitted helminth infection.
(TIF)
