# Supplementary material for: Risk profiling of soil-transmitted helminth infection and estimated number of infected people in South Asia: A systematic review and Bayesian geostatistical Analysis
Source: PLoS Negl Trop Dis. 2019 Aug 9;13(8):e0007580. doi: 10.1371/journal.pntd.0007580 (PMC6709929; doi:10.1371/journal.pntd.0007580)
Supplement: S1 Table — (DOCX) [file pntd.0007580.s002.docx]

**S1 Table. Overview of diagnostic methods of surveys, stratified by country.**

| **Countries** | | **Bangladesh** | **India** | **Nepal** | **Pakistan** | **Total** |
| --- | --- | --- | --- | --- | --- | --- |
|  | | ***A. lumbricoides*** | | | | |
| Diagnostic methods (number & percentage of surveys)^a^ | KK | 4 (2.99) | 113 (37.54) | 23 (30.67) | 4 (15.38) | 144 (26.87) |
|  | Mini-FLOTAC | 99 (73.88) | 0 (0.00) | 0 (0.00) | 0 (0.00) | 99 (18.47) |
|  | FEC | 7 (5.22) | 38 (12.62) | 18 (24.00) | 5 (19.23) | 68 (12.69) |
|  | SF | 1 (0.75) | 16 (5.32) | 0 (0.00) | 1 (3.85) | 18 (3.36) |
|  | DS | 8 (5.97) | 22 (7.31) | 17 (22.67) | 6 (23.08) | 53 (9.89) |
|  | Combine | 5 (3.73) | 80 (26.58) | 6 (8.00) | 9 (34.62) | 100 (18.66) |
|  | Other | 6 (4.48) | 4 (1.33) | 0 (0.00) | 0 (0.00) | 10 (1.87) |
|  | NS | 4 (2.99) | 28 (9.30) | 11 (14.67) | 1 (3.85) | 44 (8.21) |
| Missing information (number & percentage of surveys)^b^ | | 115 (85.82) | 200 (66.45) | 55 (73.33) | 21 (80.77) | 391 (72.95) |
|  | | ***T. trichiura*** | | | | |
| Diagnostic methods (number & percentage of surveys)^a^ | KK | 4 (3.10) | 87 (43.72) | 24 (34.78) | 1 (7.69) | 116 (28.29) |
|  | Mini-FLOTAC | 99 (76.74) | 0 (0.00) | 0 (0.00) | 0 (0.00) | 99 (24.15) |
|  | FEC | 6 (4.65) | 24 (12.06) | 17 (24.64) | 4 (30.77) | 51 (12.44) |
|  | SF | 1 (0.78) | 12 (6.03) | 0 (0.00) | 1 (7.69) | 14 (3.41) |
|  | DS | 6 (4.65) | 12 (6.03) | 13 (18.84) | 5 (38.46) | 36 (8.78) |
|  | Combine | 5 (3.88) | 39 (19.60) | 5 (7.25) | 2 (15.38) | 51 (12.44) |
|  | Other | 5 (3.88) | 3 (1.51) | 0 (0.00) | 0 (0.00) | 8 (1.95) |
|  | NS | 3 (2.33) | 22 (11.06) | 10 (14.49) | 0 (0.00) | 35 (8.54) |
| Missing information (number & percentage of surveys)^b^ | | 113 (87.60) | 155 (77.89) | 51 (73.91) | 9 (69.23) | 328 (80.00) |
|  | | **Hookworm** | | | | |
| Diagnostic methods (number & percentage of surveys)^a^ | KK | 2 (1.55) | 101 (35.56) | 23 (35.38) | 0 (0.00) | 126 (25.71) |
|  | Mini-FLOTAC | 99 (76.74) | 0 (0.00) | 0 (0.00) | 0 (0.00) | 99 (20.20) |
|  | FEC | 8 (6.20) | 40 (14.08) | 16 (24.62) | 4 (33.33) | 68 (13.88) |
|  | SF | 1 (0.78) | 25 (8.80) | 0 (0.00) | 1 (8.33) | 27 (5.51) |
|  | DS | 7 (5.43) | 15 (5.28) | 14 (21.54) | 5 (41.67) | 41 (8.37) |
|  | Combine | 5 (3.88) | 71 (25.00) | 6 (9.23) | 2 (16.67) | 84 (17.14) |
|  | Other | 4 (3.10) | 0 (0.00) | 0 (0.00) | 0 (0.00) | 4 (0.82) |
|  | NS | 3 (2.33) | 32 (11.27) | 6 (9.23) | 0 (0.00) | 41 (8.37) |
| Missing information (number & percentage of surveys)^b^ | | 114 (88.37) | 203 (71.48) | 47 (72.31) | 8 (66.67) | 372 (75.92) |

^a^KK: Kato-Katz; FEC: Formol-ether concentration; SF: stool flotation; DS: direct smear; Combine: combined with two or more diagnostic methods; Other: other methods; NS: not stated; ^b^Information on the number of stool samples or the number of slides per sample is missing.
