# Supplementary material for: Risk profiling of soil-transmitted helminth infection and estimated number of infected people in South Asia: A systematic review and Bayesian geostatistical Analysis
Source: PLoS Negl Trop Dis. 2019 Aug 9;13(8):e0007580. doi: 10.1371/journal.pntd.0007580 (PMC6709929; doi:10.1371/journal.pntd.0007580)
Supplement: S2 Table — (DOCX) [file pntd.0007580.s003.docx]

**S2 Table. Overview of soil-transmitted helminth surveys in the remaining countries (Afghanistan, Bhutan, the Maldives, and Sri Lanka) in South Asia^a^.**

| **Countries** | | | **Afghanistan** | **Bhutan** | **The Maldives** | **Sri Lanka** | **Total** |
| --- | --- | --- | --- | --- | --- | --- | --- |
| Records identified through literature search | | | 30 | 3 | 241 | 192 | 360 |
|  | | | ***A. lumbricoides*** | | | | |
| Relevant papers | | | 2 | 1 | 0 | 13 | 16 |
| Total surveys/locations | | | 5/5 | 1/1 | 0 | 27/25 | 33/31 |
| Period | | | 2003-2014 | 2003-2003 | - | 1967-2012 | 1967-2014 |
| Year of Survey (surveys/locations) | <1980 | Point | 0/0 | 0/0 | 0/0 | 1/1 | 1/1 |
|  |  | District | 0/0 | 0/0 | 0/0 | 1/1 | 1/1 |
|  |  | Adm1/region^b^ | 0/0 | 0/0 | 0/0 | 0/0 | 0/0 |
|  | 1980-2000 | Point | 0/0 | 0/0 | 0/0 | 4/4 | 4/4 |
|  |  | District | 0/0 | 0/0 | 0/0 | 3/2 | 3/2 |
|  |  | Adm1/region^b^ | 0/0 | 0/0 | 0/0 | 1/1 | 1/1 |
|  | >=2000 | Point | 1/1 | 0/0 | 0/0 | 0/0 | 1/1 |
|  |  | District | 0/0 | 0/0 | 0/0 | 7/7 | 7/7 |
|  |  | Adm1/region^b^ | 4/4 | 1/1 | 0/0 | 10/9 | 15/14 |
|  | | | ***T. trichiura*** | | | | |
| Relevant papers | | | 2 | 1 | 0 | 14 | 17 |
| Total surveys/locations | | | 5/5 | 1/1 | 0 | 27/25 | 33/31 |
| Period | | | 2003-2014 | 2003-2003 | - | 1967-2012 | 1967-2014 |
| Year of Survey (surveys/locations) | <1980 | Point | 0/0 | 0/0 | 0/0 | 1/1 | 1/1 |
|  |  | District | 0/0 | 0/0 | 0/0 | 1/1 | 1/1 |
|  |  | Adm1/region^b^ | 0/0 | 0/0 | 0/0 | 0/0 | 0/0 |
|  | 1980-2000 | Point | 0/0 | 0/0 | 0/0 | 4/4 | 4/4 |
|  |  | District | 0/0 | 0/0 | 0/0 | 4/3 | 4/3 |
|  |  | Adm1/region^b^ | 0/0 | 0/0 | 0/0 | 1/1 | 1/1 |
|  | >=2000 | Point | 1/1 | 0/0 | 0/0 | 0/0 | 1/1 |
|  |  | District | 0/0 | 0/0 | 0/0 | 7/7 | 7/7 |
|  |  | Adm1/region^b^ | 4/4 | 1/1 | 0/0 | 9/8 | 14/13 |
|  | | | **Hookworm** | | | | |
| Relevant papers | | | 2 | 1 | 0 | 13 | 16 |
| Total surveys/locations | | | 5/5 | 1/1 | 0 | 28/25 | 34/31 |
| Period | | | 2003-2014 | 2003-2003 | - | 1967-2012 | 1967-2012 |
| Year of Survey (surveys/locations) | <1980 | Point | 0/0 | 0/0 | 0/0 | 2/1 | 2/1 |
|  |  | District | 0/0 | 0/0 | 0/0 | 1/1 | 1/1 |
|  |  | Adm1/region^b^ | 0/0 | 0/0 | 0/0 | 0/0 | 0/0 |
|  | 1980-2000 | Point | 0/0 | 0/0 | 0/0 | 4/4 | 4/4 |
|  |  | District | 0/0 | 0/0 | 0/0 | 3/2 | 3/2 |
|  |  | Adm1/region^b^ | 0/0 | 0/0 | 0/0 | 1/1 | 1/1 |
|  | >=2000 | Point | 1/1 | 0/0 | 0/0 | 0/0 | 1/1 |
|  |  | District | 0/0 | 0/0 | 0/0 | 7/7 | 7/7 |
|  |  | Adm1/region^b^ | 4/4 | 1/1 | 0/0 | 10/9 | 15/14 |

^a^ The review was carried out on 24 May, 2017; ^b^surveys aggregated at administrative division of level one or at regional level.
