# Supplementary material for: Risk profiling of soil-transmitted helminth infection and estimated number of infected people in South Asia: A systematic review and Bayesian geostatistical Analysis
Source: PLoS Negl Trop Dis. 2019 Aug 9;13(8):e0007580. doi: 10.1371/journal.pntd.0007580 (PMC6709929; doi:10.1371/journal.pntd.0007580)
Supplement: S3 Table — (DOCX) [file pntd.0007580.s004.docx]

**S3 Table. Number (×10^6^) and percentage (%) of infected individuals living in areas with low, moderate, and high risk of soil-transmitted helminth infections, stratified by country^a^.**

| **Countries** | |  | **Bangladesh** | **India** | **Nepal** | **Pakistan** | **Total** |
| --- | --- | --- | --- | --- | --- | --- | --- |
| Low risk areas | Entire population | No. of  infected | 5.62 (4.91: 6.33) | 68.82 (64.07: 74.68) | 1.63 (1.42: 1.86) | 11.12 (10.17: 12.17) | 87.12 (81.25: 93.97) |
|  |  | Percentage^b^ | 9.48 (7.62: 11.33) | 26.64 (23.29: 31.25) | 18.22 (14.22: 23.76) | 32.27 (25.76: 40.02) | 24.08 (21.11: 27.84) |
|  | School-aged children | No. of  infected | 1.11 (0.97: 1.25) | 13.28 (12.37: 14.39) | 0.37 (0.32: 0.42) | 2.43 (2.23: 2.67) | 17.19 (16.07: 18.55) |
|  |  | Percentage^b^ | 9.33 (7.54: 11.25) | 26.36 (23: 30.86) | 17.97 (13.95: 23.35) | 31.95 (25.27: 39.06) | 23.85 (20.9: 27.57) |
| Moderate risk areas | Entire population | No. of  infected | 19.06 (16.9: 21.39) | 107.48 (95.37: 118.65) | 3.87 (3.33: 4.4) | 15.15 (12.2: 18.42) | 145.66 (131.12: 160.84) |
|  |  | Percentage^b^ | 31.95 (27.86: 37.15) | 41.62 (38.85: 44.43) | 43.5 (37.31: 49.41) | 43.9 (40.41: 47.65) | 40.21 (37.61: 43.14) |
|  | School-aged children | No. of  infected | 3.8 (3.37: 4.27) | 20.95 (18.7: 23.27) | 0.89 (0.77: 1) | 3.35 (2.76: 4.08) | 28.98 (26.22: 32.21) |
|  |  | Percentage^b^ | 31.77 (27.75: 37) | 41.63 (38.82: 44.5) | 43.5 (36.94: 49.42) | 43.98 (40.33: 47.7) | 40.19 (37.56: 43.16) |
| High risk areas | Entire population | No. of  infected | 34.98 (29.21: 41.09) | 81.44 (58.99: 103.19) | 3.4 (2.33: 4.76) | 8.13 (4.98: 12.7) | 128.46 (99.47: 156.08) |
|  |  | Percentage^b^ | 58.71 (52.28: 63.61) | 31.69 (25.09: 37.34) | 38.02 (29.13: 47.49) | 23.72 (16.04: 31.83) | 35.54 (29.62: 40.46) |
|  | School-aged children | No. of  infected | 7.05 (5.87: 8.21) | 16.06 (11.61: 20.18) | 0.79 (0.54: 1.1) | 1.82 (1.1: 2.83) | 25.81 (20.15: 31.27) |
|  |  | Percentage^b^ | 58.95 (52.6: 63.75) | 32.01 (25.29: 37.39) | 38.3 (29.55: 47.89) | 23.95 (16.18: 32.33) | 35.85 (29.71: 40.79) |

^a^Low, moderate and, high risk areas indicate areas with predicted prevalence <20%, between 20% and 50%, and ≥50%, respectively; ^b^equalling to number of infected population living in the corresponding risk areas divided by total number of infected population of the whole study region.
