# Supplementary material for: Risk profiling of soil-transmitted helminth infection and estimated number of infected people in South Asia: A systematic review and Bayesian geostatistical Analysis
Source: PLoS Negl Trop Dis. 2019 Aug 9;13(8):e0007580. doi: 10.1371/journal.pntd.0007580 (PMC6709929; doi:10.1371/journal.pntd.0007580)
Supplement: S4 Table — (DOCX) [file pntd.0007580.s005.docx]

**S4 Table. Posterior summaries (median and 95% Bayesian credible interval) of the model parameters, using population-weighted centroids as representative locations for district-level survey data.**

| ***A. lumbricoides*** | | | | **Estimate** |
| --- | --- | --- | --- | --- |
| Period (<1980) ^a^ | | | |  |
|  | | 1980-1999 | | 0.61 (0.50; 0.72)^b^ |
|  | | ≥2000 | | -0.00 (-0.11; 0.10) |
| Survey type (school-based)^a^ | | | |  |
|  | | Community-based | | -0.03 (-0.11; 0.06) |
| Land surface temperature in the day time (25-30°C)^a^ | | | |  |
|  | | ≤8 | | 0.05 (-3.90; 3.81) |
|  | | 8-20 | | 1.49 (0.85; 2.20)^b^ |
|  | | 20-25 | | 0.79 (0.22; 1.29)^b^ |
|  | | 30-35 | | -0.60 (-1.21; 0.07) |
|  | | >35 | | -0.77 (-1.50; -0.01)^b^ |
| Human influence index (≤22)^a^ | | | |  |
|  | 22-32 | | | 0.19 (-0.24; 0.62) |
|  | >32 | | | 0.95 (0.48; 1.51)^b^ |
| Range (km) | | | | 124.13 (77.29; 196.47) |
| Spatial variance (σ^2^_sp_) | | | | 2.07 1.39; 2.97) |
| Non-spatial variance (σ^2^_nonsp_) | | | | 1.16 (0.79; 1.63) |
| ***T. trichiura*** | | | | **Estimate** |
| Period (<1980)^a^ | | | |  |
|  | 1980-1999 | | | 1.25 (1.26; 1.54)^b^ |
|  | ≥2000 | | | 0.34 (0.19; 0.48)^b^ |
| Precipitation seasonality (90-110%)^a^ | | | |  |
|  | ≤70 | | | -0.12 (-1.06; 0.74) |
|  | 70-90 | | | 0.55 (-0.16; 1.27) |
|  | 110-130 | | | -1.01 (-1.53; -0.35)^b^ |
|  | >130 | | | -1.98 (-3.08; -0.12)^b^ |
| Land surface temperature in the day time (≤26.5°C)^a^ | | | |  |
|  | 26.5-31 | | | -0.21 (-0.89; 0.43) |
|  | >31 | | | -1.30 (-2.11; -0.45)^b^ |
| Range (km) | | | | 125.48 (65.14; 285.61) |
| Spatial variance (σ^2^_sp_) | | | | 2.16 (1.26; 3.62) |
| Non-spatial variance (σ^2^_nonsp_) | | | | 0.93 (0.49; 1.58) |
| **Hookworm** | | | | **Estimate** |
| Period (<1980)^a^ | | | |  |
|  | | | 1980-1999 | -0.59 (-0.77; -0.40)^b^ |
|  | | | ≥2000 | -0.64(-0.84; -0.44)^b^ |
| Normalized differenced vegetation index (≤0.40)^a^ | | | |  |
|  | | | 0.40-0.53 | 0.21 (-0.16; 0.69) |
|  | | | >0.53 | 0.59 (0.22; 1.06)^b^ |
| Open defecation (≤15%)^a^ | | | |  |
|  | | | 15-60 | 0.60 (0.13; 0.93)^b^ |
|  | | | >60 | 0.08 (-0.36; 0.56) |
| Range (km) | | | | 151.19 (59.70; 269.34) |
| Spatial variance (σ^2^_sp_) | | | | 2.08 (1.33; 3.27) |
| Non-spatial variance (σ^2^_nonsp_) | | | | 1.05 (0.70; 1.50) |

^a^In brackets, baseline values are reported; ^b^important effect based on 95% Bayesian credible interval (BCI).
