# Supplementary material for: Risk profiling of soil-transmitted helminth infection and estimated number of infected people in South Asia: A systematic review and Bayesian geostatistical Analysis
Source: PLoS Negl Trop Dis. 2019 Aug 9;13(8):e0007580. doi: 10.1371/journal.pntd.0007580 (PMC6709929; doi:10.1371/journal.pntd.0007580)
Supplement: S5 Table — (DOCX) [file pntd.0007580.s006.docx]

**S5 Table. Population-adjusted predicted prevalence (%) and number of individuals (×10^6^) infected by soil-transmitted helminths^a^, using population-weighted centroids as representative locations for district-level survey data.**

| **Countries** | | **Bangladesh** | **India** | **Nepal** | **Pakistan** | **Total** |
| --- | --- | --- | --- | --- | --- | --- |
| *A. lumbricoides* | Prevalence | 21.64 (18.04; 25.16) | 12.31 (10.31; 14.24) | 15.12 (12.21; 18.99) | 11.31 (8.49; 14.69) | 13.14 (11.23; 15.02) |
|  | No. of entire population infected | 34.04 (28.39; 39.6) | 154.86 (129.8; 179.2) | 4.94 (3.99; 6.2) | 21.35 (16.03; 27.74) | 215.12 (183.91; 246) |
|  | No. of school-aged children infected | 6.87 (5.66; 8.03) | 30.26 (25.63; 35.03) | 1.15 (0.92; 1.42) | 4.77 (3.54; 6.19) | 43.16 (37.07; 48.86) |
| *T. trichiura* | Prevalence | 19.08 (15.69; 22.58) | 3.23 (2.38; 4.09) | 7.13 (5.38; 10.03) | 3.55 (2.24; 5.49) | 4.91 (3.98; 5.85) |
|  | No. of entire population infected | 30.03 (24.68; 35.53) | 40.61 (29.93; 51.52) | 2.33 (1.76; 3.28) | 6.7 (4.23; 10.36) | 80.33 (65.17; 95.85) |
|  | No. of school-aged children infected | 5.98 (4.92; 7.08) | 7.86 (5.79; 9.97) | 0.53 (0.4; 0.75) | 1.47 (0.93; 2.27) | 15.98 (12.96; 19.12) |
| Hookworm | Prevalence | 8.75 (6.87; 10.87) | 8.23 (6.47; 10.15) | 10.93 (8.57; 14.47) | 5.39 (3.03; 8.02) | 7.99 (6.45; 9.85) |
|  | No. of entire population infected | 13.76 (10.82; 17.11) | 103.56 (81.37; 127.78) | 3.57 (2.8; 4.73) | 10.18 (5.72; 15.13) | 130.81 (105.54; 161.23) |
|  | No. of school-aged children infected | 2.74 (2.16; 3.41) | 20.04 (15.75; 24.73) | 0.81 (0.64; 1.08) | 2.23 (1.26; 3.32) | 25.78 (20.78; 31.81) |
| Any soil-transmitted helminth | Prevalence | 37.88 (34.33; 41.33) | 20.53 (18.34; 22.97) | 27.69 (24.39; 31.83) | 18.02 (14.48; 21.85) | 21.95 (19.88; 24.33) |
|  | No. of entire population infected | 59.61 (54.02; 65.03) | 258.36 (230.86; 289.11) | 9.05 (7.97; 10.4) | 34.02 (27.34; 41.25) | 359.35 (325.58; 398.34) |
|  | No. of school-aged children infected | 11.94 (10.77; 13.03) | 50.1 (44.81; 56.5) | 2.07 (1.85; 2.37) | 7.52 (6.11; 9.14) | 71.51 (64.89; 79.32) |

^a^Estimates were based on gridded population of 2015; calculations were based on the median and 95% BIC of the posterior predictive distribution of the infection risk from 2000 onwards.
