# Supplementary figures and images for: Risk profiling of soil-transmitted helminth infection and estimated number of infected people in South Asia: A systematic review and Bayesian geostatistical Analysis
Source: PLoS Negl Trop Dis. 2019 Aug 9;13(8):e0007580. doi: 10.1371/journal.pntd.0007580 (PMC6709929; doi:10.1371/journal.pntd.0007580)

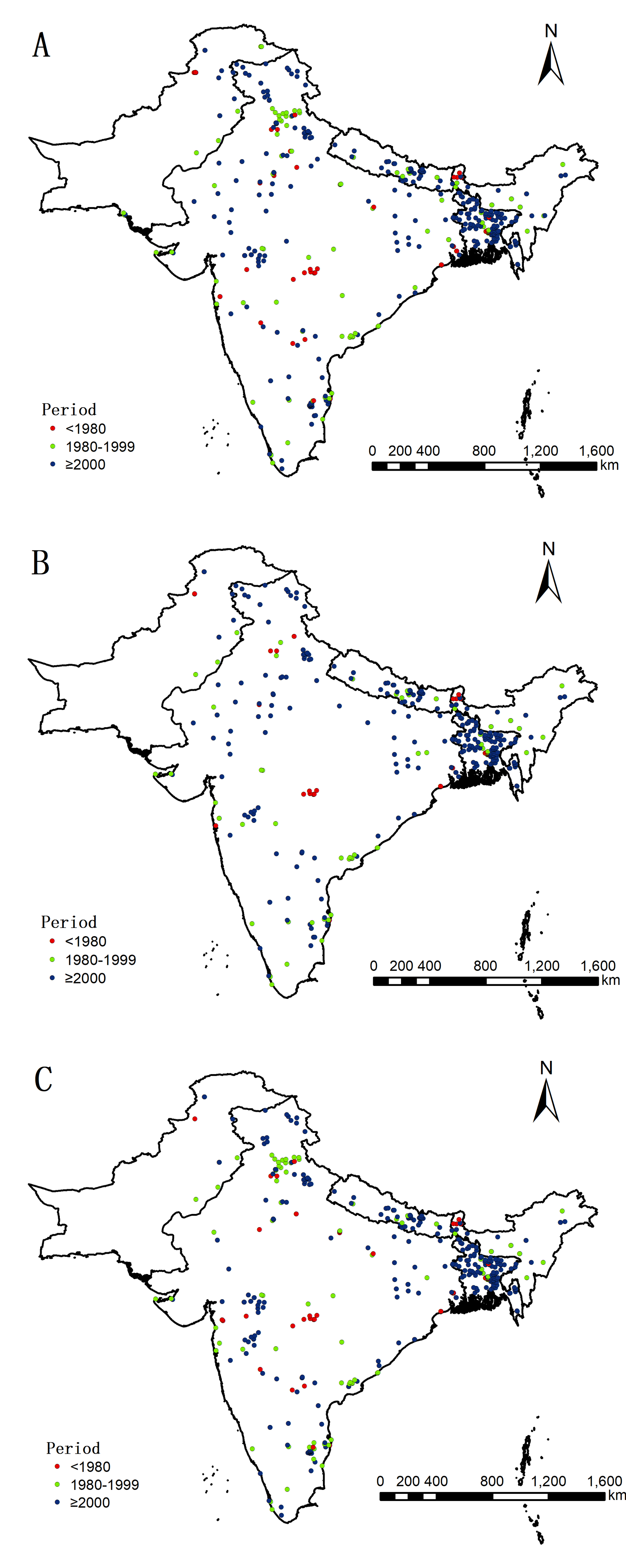

Supplement: S1 Fig — (A) A. lumbricoides, (B) T. trichiura, and (C) hookworm. (TIF) [file pntd.0007580.s007.tif]

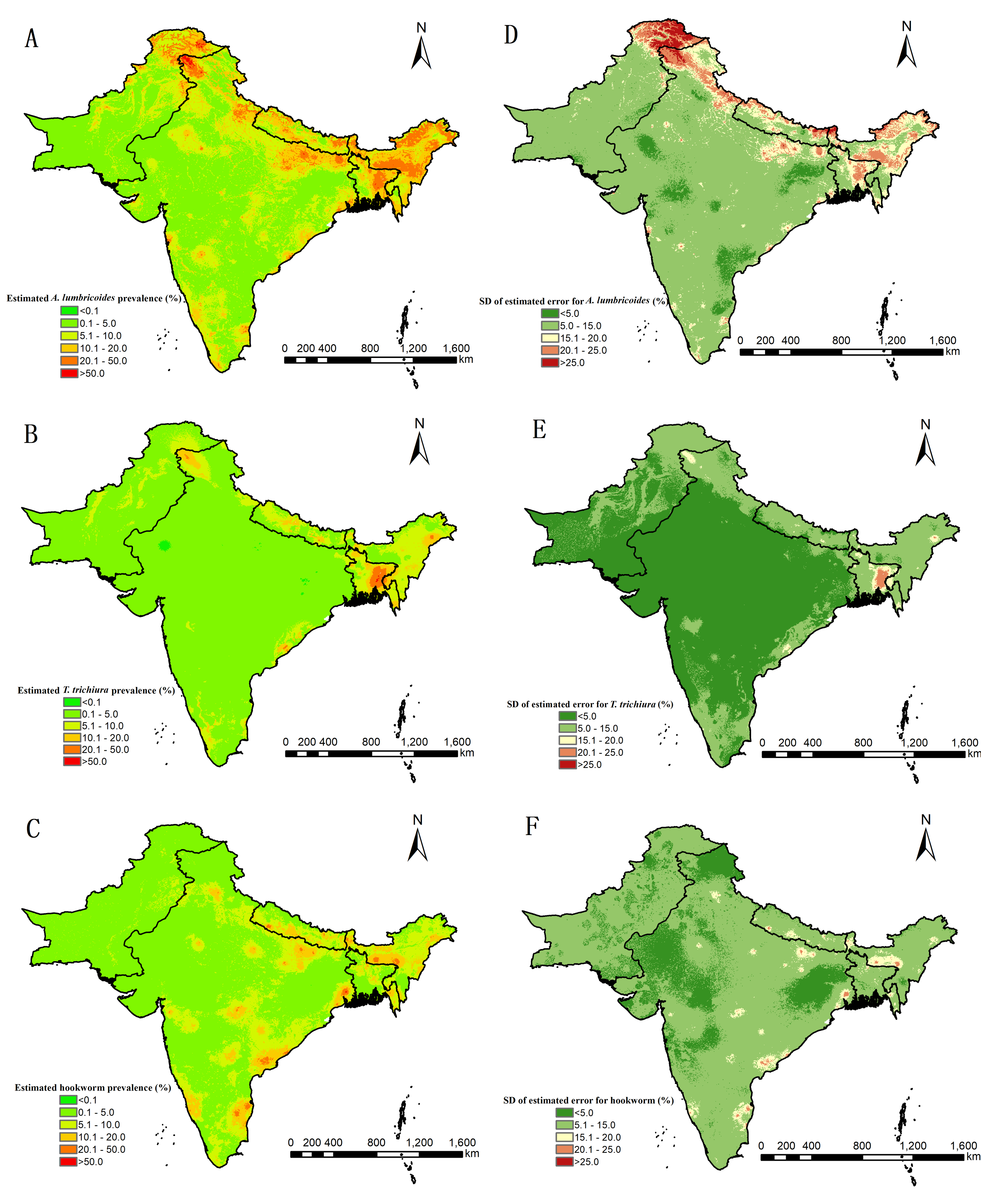

Supplement: S2 Fig — Predictive prevalence based on the median of the posterior predictive distribution of infection risk for (A) A. lumbricoides, (B) T. trichiura, (C) hookworm, and (D) any soil-transmitted helminth infection. Prediction uncertainty based on the standard deviation of the posterior predictive distribution of infection risk for (E) A. lumbricoides, (F) T. trichiura, (G) hookworm, and (H) any soil-transmitted helminth infection. (TIF) [file pntd.0007580.s008.tif]
